# Recent Advances in HBV Reactivation Research

**DOI:** 10.1155/2018/2931402

**Published:** 2018-12-26

**Authors:** Lixia Guo, Dan Wang, Xiping Ouyang, Ni Tang, Xuemei Chen, Yuhong Zhang, Hongquan Zhu, Xiaosong Li

**Affiliations:** ^1^Department of Clinical Laboratory, The Second Hospital of Jilin University, Changchun 130041, China; ^2^Department of Clinical Laboratory, The People's Hospital of Rongchang District, Chongqing 402460, China; ^3^Department of Obstetrics and Gynecology, The First Affiliated Hospital of Chongqing Medical University, Chongqing 400016, China; ^4^Key Laboratory of Molecular Biology for Infectious Diseases (Ministry of Education), Chongqing Medical University, Chongqing 400016, China; ^5^Clinical Molecular Medicine Testing Center, The First Affiliated Hospital of Chongqing Medical University, Chongqing 400016, China

## Abstract

Hepatitis B virus (HBV) is an important public health problem that poses a serious threat to human health. HBV reactivation generally occurs in overt or occult HBV infection patients who suffered DDAs, chemotherapy, or immunosuppressive therapy, especially when some solid tumors and leukemia patients are using hormones such as prednisolone and imatinib. The approximate incidence of HBV reactivation ranged from about 10% to 40%. Scientists often explore the molecular mechanisms from both the virus and the host. But some studies have reported that some drugs (cisplatin, rituximab, imatinib, and glucocorticoid) could induce HBV reactivation directly. However, the specific molecular mechanisms were unclear. With the emergence of new antiviral drugs and molecular targeted drugs, the risk of HBV reactivation will increase significantly. Therefore this review was expected to be used to provide recommendations for future research in HBV reactivation.

## 1. Introduction

**Figure 1:**
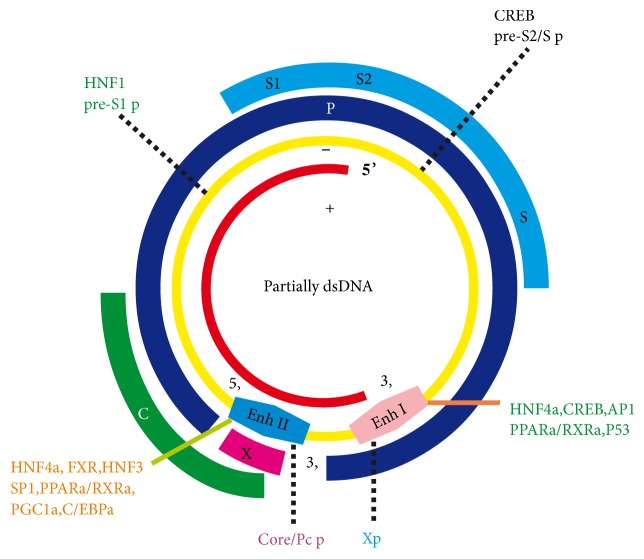
HBV binds metabolic-related transcription factors to its genome to activate its transcription.

HBV is a partially double-stranded circular DNA (3.2 kb) that belongs to the orthohepadnavirus of the Hepadnaviridae family which could infect hepatocytes and lead to liver pathologic changes. The HBV genome contains four overlapping open reading frames which could encode HBV polymerase, HBsAg, HBx, and HBeAg protein. Basically, the HBV gene expression is controlled at the transcriptional level by two enhancers and four promoters (Figure 1) [1].

Hepatitis B virus (HBV) is an important public health problem that poses a serious threat to human health. According to the World Health Organization, two billion people worldwide have been infected with HBV, and about 400 million of them have become chronic carriers that hepatitis B virus surface antigens (HBsAg) are positive, and 3/4 of them are from China. In addition, nearly one million people died because of HBV infection every year in the world, half of them in China [2]. At the same time, according to the statistics of the World Health Organization, the main cause of human deaths is still malignant tumors. The number of cancer patients with HBV infection is relatively large [3], yet usually, HBV reactivation is rarely happened in patients with chronic HBV infection and asymptomatic HBV carriers. However, when these patients receive chemotherapy or immunosuppressive therapy, HBV in a resting state or a low replication state is likely to be transiently activated, which would lead to severe liver function damage or even liver failure. It may lead to slowing or interruption of treatment conversely, and then delaying the effective treatment of cancer patients that would seriously affect the prognosis, some of which may even be life-threatening [4–6].

At present, although there are no absolute unified diagnostic criteria for HBV reactivation at home and abroad, basically a consensus has been reached [7, 8]: it is usually based on clinical experience that when a tumor patient or an organ transplant recipient is receiving chemotherapy or immunosuppressive therapy, there is a sudden increase in serum hepatitis B virus DNA (HBV DNA) levels that occur at least 10 times (relative to baseline) or its absolute value was more than 10^9^ copies/mL, or accompanied by alanine aminotransferase (ALT), aspartate aminotransferase (AST) levels increased by at least 3 times could be called HBV reactivation. It is also clearly defined as the characteristic category of HBV reactivation when the HBV DNA copy number in the serum of some asymptomatic carriers (cured or inactive HBV infection) sharply increases. Such phenomenon is a process that often occurs with the disease but not always. HBV reactivation could occur spontaneously according to the state of the body, but it happened mostly in cancer patients after chemotherapy or immunosuppressive therapy which would lead to acute hepatitis, acute severe hepatitis, and even acute liver failure frequently. However, a large number of HBV reactivation occurred in some specific subclinical cases, such as occult infections of HBV which was infected by sexual or family contact, and the HBV reactivation is highly prevalent when he or she is in the late stage of liver disease. At the same time, the patients could receive the preintervention treatment with antiviral drugs when they have the risk of HBV reactivation. However, the patients who should require antiviral therapy and the time, dose, and duration of treatment are still unclear. HBV reactivation reveals its complex virological characteristics and irresistibility of occult infections; although HBV DNA is not clinically monitored, the viral genome could be bound to the DNA of the patient's hepatocyte nucleus, thus triggering the reactivation of HBV under specific conditions, which then could cause a series of serious clinical symptoms. Nothing is more important, many clinicians mistakenly believe that this phenomenon is a superposition of HBV latent infection and drug-induced or alcoholic hepatitis. Therefore, it is necessary to further understand the phenomenon and molecular mechanism of HBV reactivation, so as to choose the appropriate way and time to avoid the harm.

## 2. Clinical Virological Characteristics of HBV Reactivation

HBV reactivation generally occurs in some cancer patients after chemotherapy, immunosuppressive therapy, and biological modifier therapies [9], especially when some solid tumors and leukemia patients are using hormones such as prednisolone and rituximab that emerged clinical crisis. It can also occur in some patients with autoimmune diseases, organ transplants (kidney transplants, lung transplants, heart transplants, etc.) and human immunodeficiency virus (HIV), but the most serious cases are often with bone marrow or liver transplants [10–12]. At the same time, drugs such as some tyrosine kinase inhibitors [13] have a certain relationship with HBV reactivation, but the pathogenic mechanism related to HBV reactivation needs further research. Yet still, the typical course of HBV reactivation is generally recognized, of which the three phases are shown in Table 1 [14]. 

**Figure 2:**
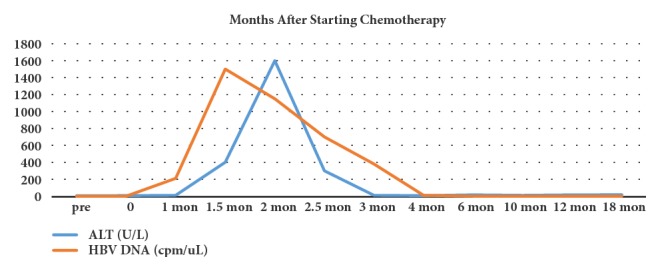
The typical course of HBV reactivation.

Phase 1 refers to the initiation of HBV reactivation following a sudden increase in HBV replication after treatment with an immunosuppressive or chemotherapeutic drug (the serum HBV DNA increase at least 10 times relative to baseline). In patients with a negative serum hepatitis B virus e antigen (HBeAg), HBeAg can be detected again in serum. Phase 2 means that when the immunosuppressant is reduced or just removed, the patient's liver cells show obvious damage and inflammation, and serum ALT level rises significantly, leading to the appearance of jaundice. And in this stage, the serum HBV DNA begins to drop. Phase 3 is the recovery period, the patient's liver cell damage is partially relieved, and the HBV DNA copy number drops to the baseline level or below. Clinically, not all patients with HBV reactivation will experience this exactly typical course; for example, in some patients, the serum HBV DNA rise several times instantaneously, but the hepatocytes and immune system of patients are not damaged obviously. Reactivation of HBV could lead to sudden or acute deterioration of hepatitis. When the patients suffered clinical treatment, the serum ALT fell below the baseline firstly, then with the continuation of treatment, the serum ALT rapidly rose above the upper limit of the baseline. If the serum ALT increases by more than 5 times compared with the upper limit of normal value, which could be called hepatitis burst, and if it increases by more than 10 times, it could be called deteriorating acute hepatitis. In the course of HBV reactivation, rise of the serum ALT level can be accompanied by that of HBV DNA level, and sometimes the HBV DNA can increase firstly, when the HBV DNA falls back, and the serum ALT level rises remarkably. Therefore, the serum ALT levels and HBV DNA levels are often used as important indicators to monitor the risk of HBV reactivation in clinical patients. The typical course of HBV reactivation is shown in Figure 2 [14].

## 3. The Epidemiological Research of HBV Reactivation 

For a long time, it was difficult for academia to accurately define the incidence of HBV reactivation; nevertheless the HBV reactivation has been a widespread phenomenon clinically. Until the 1980s, Lok ASF (a scientist who came from Hong Kong) and other researchers found that after 100 patients with lymphoma had received chemotherapy or immunosuppressive therapy, the phenomenon and occurrence of HBV reactivation were confirmed from viral serology and clinical biochemistry. Among these patients, 48% of them with serum HBsAg-positive patients developed HBV reactivation, at the same time, 4% of HBsAg-negative and anti-HBc-positive patients developed HBV reactivation after they had received chemotherapy, which was called “viral serological conversion” by the scholars and was also the most extreme mode of HBV reactivation. According to relevant research, nearly half of these patients with HBV reactivation got jaundice, and almost one in five of them would die from jaundice. Clinical data had confirmed that the incidence of HBV reactivation has been increasing in patients with solid tumors undergoing chemotherapy or immunosuppressive therapy. Nearly half of patients with hepatocellular carcinoma and hematopoietic stem cell transplants would suffer from HBV reactivation. In this group of patients, the incidence of HBV reactivation was much higher than that of acute hepatitis, which was equivalent to the incidence of drug-induced hepatocyte injury. According to relevant reports, the incidence of HBV reactivation significantly increased in patients with serum HBeAg-positive, HBV DNA high-replication levels, or treatment of glucocorticoids [15].

In fact, the serum HBV DNA levels of the vast majority of cancer patients in the clinic were not monitored before receiving chemotherapy or immunosuppressive therapy, so it was impossible to evaluate the true incidence of HBV reactivation. Consequently, the true incidence of HBV reactivation should be much higher than the reported clinically. Therefore, more and more scholars have started paying attention to clinical crisis (caused by HBV reactivation) research. At the same time, some studies [16–18] have found that, especially in patients with HBsAg-positive lymphoma, the incidence of HBV reactivation after treatment with CHOP chemotherapy (cyclophosphamide, epirubicin, vincristine, prednisone, or CHOP) had increased significantly by 30%-70%, and the mortality had also gone up. Some scholars have found that when the patients were treated with chemotherapeutic drugs such as rituximab and folinic acid, the immunity of patients would be greatly inhibited, and the incidence of HBV reactivation might also increase significantly. Rituximab could specifically bind to the CD20 antigen on the B lymphocyte membrane and then elicit an immune response to B cell lysis, which might cause serious damage to the patient. When the patients (bone marrow transplant) have received chemotherapy or immunosuppressive therapy, HBV reactivation would occur in nearly half of them. Some researches also reported that the incidence of HBV reactivation was mainly affected by the type of tumor and chemotherapy regimen.

Recently, a systematic review and meta-analysis about HBV reactivation have been reported on Hepatology [19] (an international top journal of liver diseases). From this study, it could be concluded that the proportion of HBV reactivation in chronic hepatitis C (CHC) patients who were coinfected with HBV would increase significantly, when they were treated with pan-oral direct-acting antiviral agents (DAAs). The meta-analysis showed that the pooled HBV reactivation rate was 14.1% when the CHC patients were coinfected with overt HBV, which has also been reported in other documents [20, 21]. Therefore, the European Medicine Agency's Pharmacovigilance Risk Assessment Committee (EMAPRAC) and the US Food and Drug Administration (FDA) have already confirmed the risk of HBV reactivation after DAAs therapy [22, 23]. But another retrospective study [24] has shown that only 9 of 62,290 veterans treated with oral hepatitis C antivirals (DAAs) had evidence of HBV reactivation occurring while being on DAA treatment. However, an original article [25] published recently in journal of Gastroenterology reported that the HBV reactivation had appeared often in HBsAg-positive patients during DAA therapy (63% of patients showed an increase in HBV DNA), but only 5% of patients had a concomitant increase in ALT through posttreatment week 12. Therefore, a detailed research of HBV reactivation incidence in the real world is needed to put on the agenda.

The FDA identified 29 reports of HBV reactivation in patients who have received DAAs from 22 November 2013 to 15 October 2016, and three cases resulted in death or liver transplantation [26]. So it is necessary to screen all patients if they have overt or occult HBV infection and that must be strictly managed during the treatment of pan-oral DAAs, in addition to chemotherapy drugs, the imatinib (a 2-phenyl amino pyrimidine derivative) which acts as a specific inhibitor of tyrosine kinase that would reactivate the replication of HBV, too. Therefore, the screening of hepatitis B virus should be prior to imatinib therapy [27]. This phenomenon has also been confirmed by the ruxolitinib treatment which could reactivate the replication of HBV [28], although the HBV infection could be controlled by interrupting transmission, vaccines, and antiviral treatment. The HBV subgenotype F3 which was endemic in South America but rare in any other epidemiological links to regions endemic for genotype F could reactivate with vaccine escape mutations [29]. The incidence of HBV reactivation after radiotherapy was 12.7% which was reported in a multicenter study [30]. It has been reported [31] that a patient who had already been cured of acute hepatitis B and obtained anti-HBs which was a protective antibody against HBV. However, when the patient was exposed to an immunocompromised state by using chemotherapeutic or immunosuppressive drugs, the reactivation of HBV could occur.

## 4. The Molecular Pathological Mechanism of HBV Reactivation*　　*

Although the phenomenon of HBV reactivation has been widely concerned and studied by scholars, the specific molecular mechanism of its occurrence is still not clear, it is still a thorny problem that plagues clinicians. In recent years, there have been many clinical experiments at home and abroad to confirm that cytotoxic chemotherapeutic drugs can induce HBV reactivation (the HBV DNA increased significantly), accompanied by acute liver function injury or crisis of liver failure which would lead to a sharp increase in mortality. However, the specific molecular mechanism of HBV reactivation which was induced by cytotoxic chemotherapy drugs remains unclear. At present, it is agreed at home and abroad that the occurrence and prognosis of HBV reactivation are mainly determined by two factors: host (vivo immunity) and virus (genotype). Cytotoxic chemotherapeutic drugs might interfere with the host's immune regulation of HBV replication, and the body's immunity is a key factor in controlling viral infection and replication. When the body's immunity is inhibited, viral replication will increase accordingly. As reported in these articles [32, 33], when these drugs (chemotherapeutic drugs) were given, the lymphocyte function might be suppressed and many effector pathways, including the production of viral inhibitory cytokines such as tumor necrosis factor-alpha and gamma-interferon, were inhibited. The viral peptide-expressing hepatocytes could be recognized by the cytotoxic T cells and then cause variable degrees of hepatocyte injury and necrosis. Cytotoxic chemotherapeutic drugs, such as hormonal drugs (prednisone, etc.) could directly enhance replication of HBV or induce HBV replication by activating the glucocorticoid action elements (located on the HBV genome), which in turn increase the risk of HBV reactivation.

At present, the antineoplastic drugs that cause HBV reactivation in clinic are mainly divided into two categories: one is traditional cytotoxic chemotherapy drugs, and the other is a classic biological agent related to anti-T and B lymphocyte monoclonal antibodies. These drugs have strong immunosuppressive effects, and the body's immunity is the most important factor in inhibiting viral infection and viral replication. When the body's immunity is inhibited, the level of viral replication is significantly enhanced. As reported in this literature [34], the risk of HBV reactivation seems to be associated with the intensity of immunosuppression. When a tumor patient or an organ transplant patient is treated with a chemotherapeutic drug or an immunosuppressive agent, the body's immune system will be inhibited, especially the function of T lymphocytes is significantly reduced, and the process is divided into two stages artificially. The first stage is the application of chemotherapeutic drugs, which may lead to the inhibition of the patient's immune system, especially the inhibition of T lymphocyte function, which may result in enhanced replication of HBV, and the number of HBV-infected liver cells will increase significantly, and redetection of HBV DNA in serum or a significant increase of DNA level can be seen from patients' clinical manifestation, and serological conversion of HBeAg. The second stage is the discontinuation of cytotoxic chemotherapy drugs, in this stage, the infected HBV hepatocytes will be attacked by the recovery of immune function of T lymphocytes, which may result in acute damage of hepatocyte. The clinical manifestations are acute hepatitis, acute severe hepatitis, and acute liver failure. As a result, the patient is totally devastated.

Some scholars have confirmed that when the cells (HepG2.2.15, which were cultured in vitro, could express HBV stably) are stimulated with anthracyclines, the level of HBV DNA is significantly upregulated, and there is a dose-dependent relationship between them. Some literature [35, 36] had reported that interferon- (IFN-) mediated e antigen seroconversion had a significantly higher probability of HBV reactivation than spontaneous e antigen seroconversion, especially in patients over 30 years (when HBeAg seroconversion happened), and the molecular mechanism might be related to IFN-induced gene mutation. At the same time, some studies have found that the 1762/1764 nucleotide mutation occurred in the BCP region and the point mutation at the 1896 site in the anterior core region might be related to HBV reactivation [37]. A large number of studies have found that the incidence of HBV reactivation in different genotypes also varies, and the difference is statistically significant (P<0.05); it is closely related to the titer of serum HBV DNA level and the emergence of HBeAg [38, 39]. Similar research has been reported in another literature [40]; HBV S gene immune-escape mutants were frequently found in the HBsAg-negative reactivation patients during or after immunosuppressive or cytotoxic chemotherapy, so the molecular mechanism of HBV reactivation might have a certain correlation with HBV S region variants. It is reported that people with viral loads between 1,000 and 2,000 IU/mL have the highest risk of HBV reactivation, and then the persistence of covalently closed circular DNA (cccDNA) might be a life-long risk for HBV reactivation [41].

**Figure 3:**
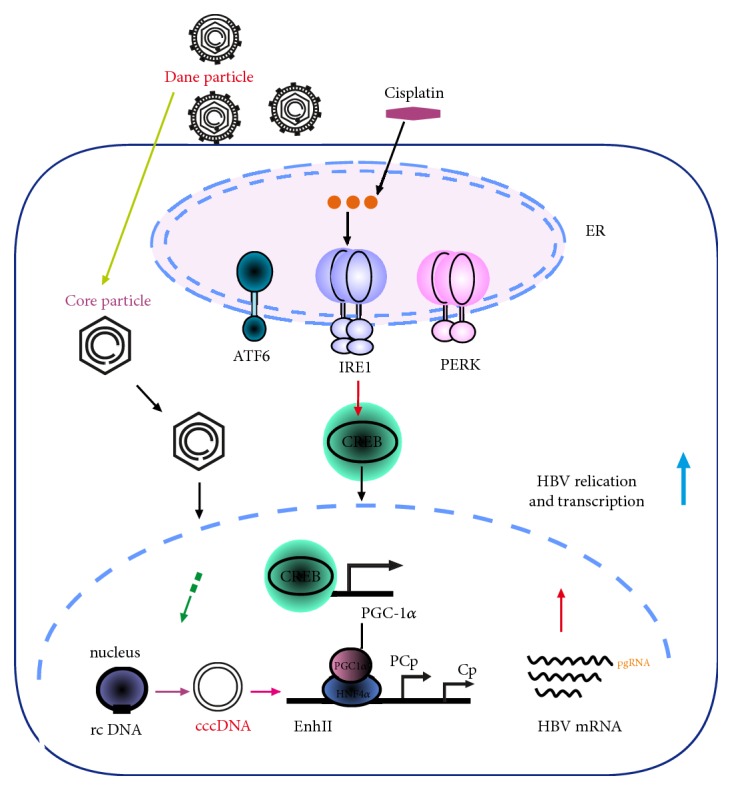
The molecular mechanism of HBV reactivation induced by cisplatin.

Based on the latest research findings [42] of our research group (Figure 3), the peroxisome proliferator-activated receptor gamma coactivator 1 alpha (PGC-1*α*) and hepatocyte nuclear factor 4 alpha (HNF-4*α*) plays a key role in cisplatin-induced HBV reactivation. Additionally, the upregulation of PGC-1*α* is depended on cisplatin-mediated endoplasmic reticulum (ER) stress. These findings demonstrate a novel molecular mechanism that ER stress-PGC1*α* signaling pathway plays a central role in cisplatin-evoked HBV reactivation.

**Figure 4:**
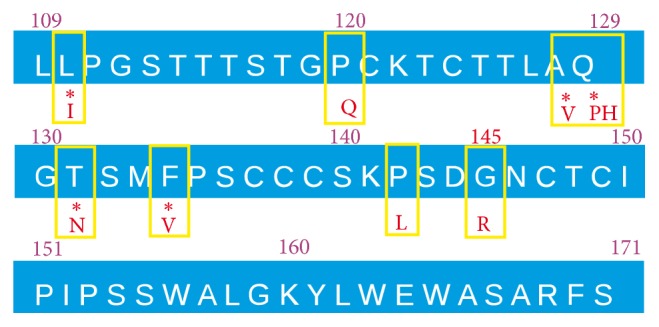
Vaccine escape mutations in the patient's hepatitis B virus strain.

Recent research has shown that the HBV subgenotype F3 could reactivate with vaccine escape mutations that there have been eight mutations and amino acid substitutions in the S protein, including the most common vaccine escape mutation G145R (Figure 4) [29].

## 5. The Prevention and Treatment of HBV Reactivation

In recent years, nucleoside analogues on the prevention and treatment of HBV reactivation have become a hot topic, and many studies have confirmed that it has good effects [43, 44]. Clinically, if the patients at the risk of HBV reactivation received prophylactic drug, its effect is significantly more effective than patients who start taking the drug after elevated serum HBV DNA. However, there are some researches also report that even after the use of preventive intervention drugs, there is no significant difference in intervening the risk of HBV reactivation in some AIDS patients and solid tumors or bone marrow transplant patients, but the overall incidence is relatively rare. The risk of HBV reactivation for prechemotherapy or immunosuppressive therapy patients should be assessed, in order to use prophylactic drug to intervene HBV reactivation. At present, the HBV reactivation risk assessment has been attracting the attention of clinicians [45, 46]. The prophylactic antiviral therapy has been recommended to patients by guidelines of HBV management [7, 47, 48].

An official recommendations [41] of the treatment of chronic hepatitis B (CHB) virus (HBV) infection in adults and children was presented on the American Association for the Study of Liver Diseases (AASLD). And according to the HBV treatment guidelines AASLD, HBV DNA >2,000 IU/mL should be considered for HBV treatment immediately. Up to now, five nucleoside analogues (entecavir, lamivudine, adefovir, ivivudine, and tenofovir) have been clinically approved to be used for treating chronic HBV infection, and lamivudine (LAM), entecavir (ETV), tenofovir (TDF), or tenofovir alafenamide (TAF) has been proved to be effective for the prevention of HBV reactivation [7, 45, 49]. The incidence rate of HBV reactivation and HBV- hepatitis might decrease by 80%-100% which was prevented with lamivudine, and the HBV liver failure might be eliminated [32]. However, long-term use of lamivudine may lead to the risk of HBV resistance mutations. An international top magazine (JAMA) has reported that the newer agents (entecavir, tenofovir disoproxil, and tenofovir alafenamide) may be associated with a significantly reduced risk of drug resistance compared with older agents (lamivudine and adefovir) and which should be considered as the first-line treatment [50]. At the same time, there are some key problems in the prophylactic use of nucleoside analogues. Who need prophylactic administration? When do patients start using preventive intervention drugs? How much are the drug dose and continuous administration time? How about the tolerance and effectiveness of patient? The systematic clinical research should be launched to resolve the above problems.

**Figure 5:**
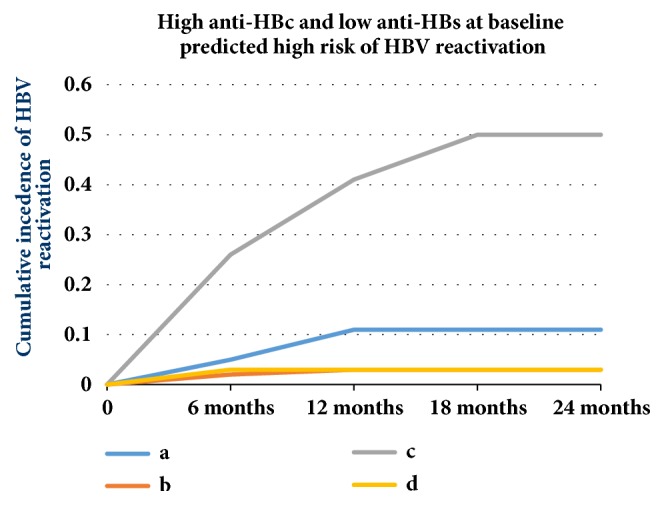
High anti-HBc and low anti-HBs at baseline predicted high risk of HBV reactivation (a: anti-HBc <6.41, anti-HBs<56.48, b: Anti-HBc <6.41, anti-HBs ≥56.48, c: Anti-HBc ≥6.41, anti-HBs<56.48, d: Anti-HBc ≥6.41, anti-HBs ≥56.48, p value ≤0.0001 (Long-rank)).

Recently, a study published online showed that the quantification of anti-HBc/anti-HBs might help predict HBV reactivation in these patients with lymphoma and resolved HBV infection when they received rituximab-containing chemotherapy, and the risk of HBV reactivation for patients with lymphoma and resolved HBV infection varied; high anti-HBc and low anti-HBs at baseline predicted high risk of HBV reactivation (Figure 5) [51]. HBV screening in patients should include the routine use of anti-HBs, and those who are anti-HBs negative should receive antiviral prophylaxis [52].

So as recommended by the US Food and Drug Administration [53], all the patients who were undergoing chemotherapy, solid organ transplantation, immunosuppressive therapy, or HSCT should be screened for active or prior HBV infection by testing for anti-HBc and HBsAg in serum. In HBsAg-positive patients with risk factors, life-long surveillance for HCC with abdominal ultrasound examination at 6-month intervals and alpha-fetoprotein (AFP) testing is required [54].

## 6. The Prospects of HBV Reactivation

The complexity of the HBV reactivation is mainly around the morbidity, pathogenesis, treatment, prevention, etc. Currently, there is still no effective solution to resolve the worldwide problem of HBV reactivation, nor a uniform treatment or prevention standard to evaluate HBV reactivation systematically. HBV reactivation is mainly related to viral factors, host factors, and the choice of chemotherapy drug regimens, which increases the difficulty of prevention and treatment of HBV reactivation. At present, in order to analyze the specific molecular mechanism of HBV reactivation, some scholars have carried out a series of experiments in vitro and in vivo for HBV reactivation, in order to find out the targeted drugs which could inhibit HBV reactivation effectively and then completely resolve the public health problems which always threaten humanity. At present, some scientists have verified the phenomenon that chemotherapeutic drugs could promote HBV DNA replication directly in vivo and in vitro simultaneously and then determine the relevant signaling pathway of this phenomenon, which could help analyze the specific molecular mechanism of HBV reactivation. For example, scientists have found the corresponding functional elements of glucocorticoid in HBV DNA and then explored the target drugs to inhibit HBV reactivation effectively, in order to analyze the specific molecular mechanism of HBV reactivation which was induced by chemotherapeutic drugs or immune inhibitor and so on, finding the new ideas and new methods to resolve HBV reactivation fundamentally. Following from that, the crisis caused by HBV reactivation could be prevented, such as acute hepatitis, acute severe hepatitis, acute liver failure, etc. It also may prevent and improve the prognosis of patients effectively, especially those who need long-term or even life-long immunosuppression therapy and chemotherapy. In addition, the patient not only can receive multiple courses of immunosuppression therapy or chemotherapy, but also can prolong the lives and improve their quality of life.

## Figures and Tables

**Table 1:** Three phases of HBV Reactivation.

**PHASE**	**FEATURE**	**DIAGNOSTIC MARKERS**	**COMMENTS**
**1**	Increase in HBV Replication Period	HBV DNA HBeAg HBsAg	Rise of > 1 log_10_ IU/mL In HBeAg negative Reverse seroconversion

**2**	Liver Disease Activity Period	ALT Jaundice Symptoms	Rise of > 3 times baseline Injury Indicates more serious

**3**	Recovery Period	HBV DNA ALT HBsAg	Drops to the baseline level Drops to the baseline level May be negative late
